# Update on tick-borne pathogens detection methods within ticks

**DOI:** 10.1016/j.crpvbd.2024.100199

**Published:** 2024-07-03

**Authors:** Eva Krupa, Alexis Dziedziech, Richard Paul, Sarah Bonnet

**Affiliations:** a Institut Pasteur, Université Paris Cité, CNRS UMR 2000, INRAE USC 1510, Ecology and Emergence of Arthropod-borne Pathogens Unit, F-75015, Paris, France; b Department of Laboratory Medicine, Lund University, Lund, Sweden

**Keywords:** Ticks, Tick-borne pathogens, Detection methods

## Abstract

Ticks are known vectors of various pathogens, including bacteria, parasites and viruses, that impact both animal and human health. Improving knowledge of the distribution of tick-borne pathogens, combined with their early detection in ticks, are essential steps to fight against tick-borne diseases and mitigate their impacts. Here we give an overview of what are the common methods of pathogen detection in tick samples, including recent developments concerning how to handle tick samples, get access to tick-borne pathogens by chemical or physical disruption of the ticks, and methods used for the RNA/DNA extraction steps. Furthermore, we discuss promising tools that are developed for other sample types such as serum or blood to detect tick-borne pathogens, and those that could be used in the future for tick samples.

## Highlights

• Review of optimal storage conditions, cleaning steps, and nucleic acid extraction of pathogens in tick samples.
• Review of the state-of-the-art of molecular diagnostics for tick-borne pathogen detection in tick samples.
• Promising tools for use in field settings, especially in resource-limited sites.

## Graphical abstract

**Figure:**
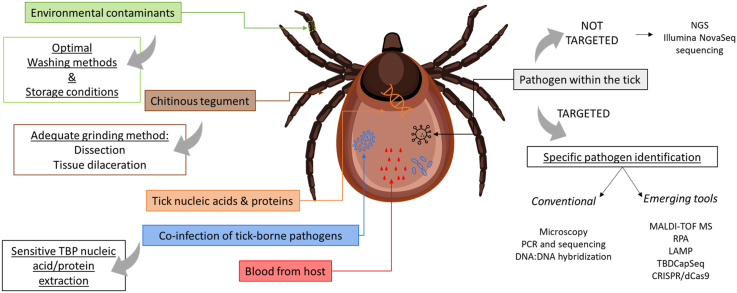

## 1 Introduction

Ticks are hematophagous acarians considered as the first vector of medical and veterinary importance in the Northern Hemisphere. They have a high economic impact on veterinary health and cause a high burden in human health by the pathogens they can transmit (Moraga-Fernández et al., 2023). Ticks can carry a high diversity of pathogens including bacteria such as *Borrelia burgdorferi* (*sensu lato*), the causative agent of Lyme disease, viruses such as tick-borne encephalitis virus or parasites such as *Babesia* spp. To date, both environmental - climate, host density, urbanisation, greening - and socio-economical changes modify the distribution and activity periods of ticks, leading to more animals and people at risk of tick bites and tick-borne diseases (TBD) (Stachurski et al., 2021).

To assess the risk of tick-borne pathogen (TBP) transmission and for the surveillance of TBD, most studies focus on pathogen detection in field-collected ticks (Díaz-Sánchez et al., 2023). Ticks are collected either by dragging or flagging methods, which involves a white sheet or flag dragged on the vegetation to collect unfed questing ticks seeking a host when they are exophilic (Dantas-Torres et al., 2013). Alternatively, unfed or engorged ticks at various stages of repletion can be collected directly on host, which represents the major way of collecting “hunting ticks” that do not quest for hosts. Tick collection methods will thus influence the species and the life-stage of ticks collected and should be adapted to the research question.

The presence of pathogens in tick haemolymph or in certain tissues can be observed under the microscope after staining. However, this is very time consuming, can present a lack of sensitivity, and requires a lot of experience, even with the use of fluorescent antibody markings, which also often present a specificity problem. Molecular detection is thus preferred due to faster results, being less dependent on the experimenter and enabling better specificity, and is now therefore considered as the gold standard method for pathogen detection in ticks. Pathogen detection in ticks by molecular methods can be done either focused on a restricted list of TBPs or realised without any *a priori*. While *a priori* methods are widely used, offering robust results across a wide range of TBPs, techniques without *a priori* offer the possibility to detect novel pathogens, either undescribed species or strains or known pathogens in areas with no previous reports.

This review discusses the different steps of pathogen detection in ticks from the storage conditions of the tick samples to nucleic acid extraction and identification of the microorganism. The various molecular techniques used for TBP detection in ticks are presented with a focus on novel ones already used or under development, and the promising tools that could be used in the future.

## 2 Storage conditions and cleaning steps

Coming from the environment, environmental contaminants can stick on ticks cuticula and may interfere with the detection of TBP. Washing ticks coming from the field with PBS, sterile water or ethanol has been tested and recently 5% sodium hypochlorite has been shown to be the preferred technique to wash ticks when internal microorganisms are studied (Binetruy et al., 2019; Hoffmann et al., 2020). However, a recent study showed that bleach interfered with female tick internal microbiota (Fernández-Ruiz et al., 2023), suggesting further investigations for a standard decontamination protocol are still needed.

While working on fresh ticks is still the best, it is not always feasible, and the storage conditions and length have an impact on the quantity and quality of nucleic acids obtained. Mtambo et al. (2006) have shown that dried preservation cannot be used, while cryopreservation in liquid nitrogen for two years and ethanol storage for 10 years give similar results on DNA extraction. For RNA extraction, ticks can be stored for short periods in RNAlater™ and then frozen at −80 °C or in liquid nitrogen (Ortiz-Baez et al., 2023). Engorged ticks, due to their large size, need more time for soaking in liquid used for storage and the volume needs to be adjusted. As both ethanol and RNAlater™ can inhibit further polymerase chain reaction (PCR) DNA amplification, they should then be removed before the extraction steps (Ammazzalorso et al., 2015).

## 3 Disruption of ticks

The first step to detect any pathogen is to disrupt tick tissues and, from this stage, ticks can be processed individually or pooled in batches. The tegument of ticks is made of chitin, which can be especially hard to break. In addition, digested blood from engorged ticks collected from hosts can clot and thus become harder to break, or interfere with lysing buffer, resulting in poor lysing. With the lack of scutellum, soft tick teguments are easier to break than in hard ticks, and in that case, chemical lysing using Proteinase K can be used for DNA extraction (Cafiso et al., 2016). For hard ticks, physical disruption is preferably used as it is more efficient, especially for the adult stage (Halos et al., 2004).

Dissection in 2 or 4 parts using a scalpel can be used alone or prior to another crushing method before the nucleic acid extraction (Ammazzalorso et al., 2015). These manual methods can be performed without training or special equipment but require sterilization of the material between samples to avoid cross-contamination, is difficult for small samples such as larvae, and is time consuming (Crowder et al., 2010; Jones et al., 2020). Tissue dilaceration can be also performed by manual crushing using a piston pellet (Bhatia and Baersch, 2024; Ghodrati et al., 2024), or crushing with liquid nitrogen in a mini-mortar (Orkun et al., 2014). Mechanical methods where the disruption is caused by the high-speed shaking of ticks and beads together, with or without the addition of liquid are also widely used. Combination of various beads (silica, sterile sand, zirconium, solid stainless steel, tungsten carbide beads, gold plated tungsten hollow core beads) and various bead beater devices have been explored (Halos et al., 2004; Crowder et al., 2010; Kato and Mayer, 2013; Jones et al., 2020; Intirach et al., 2024). Use of beads is a more reliable method, and several authors have shown that high quantity and quality of nucleic acids can be obtained through bead-beating methods (Crowder et al., 2010; Ammazzalorso et al., 2015; Jones et al., 2020). Of note, some type of beads like zirconium-silica or steel beads can inhibit PCR and extended duration of bead-beating can result in the degradation of ribosomal RNA bands, so specific bead-beating program should be followed (Crowder et al., 2010). Several commercialized tissue homogenizers were assessed, and Jones et al. (2020) showed that Beads ruptor 24 Elite results in the most constant disruption for *Amblyomma americanum*, followed by Precellys 24 and Gentle MACS Dissociation, despite the fact that DNA subsequently extracted was of enough quality for analysis in all these tested devices. Mechanical methods have the benefits of consistency, reproducibility, time efficiency, and nucleic acid extraction efficiency compared to manual methods. However, the cost and power supply for bead-beating devices limits their use to dedicated laboratories.

## 4 Nucleic acids extraction methods

After disruption, nucleic acids can be extracted by various methods, according to the targeted TBP. In most cases, DNA is used for bacteria and parasite detection. However, the detection of pathogen DNA in a tick is not a proof of the viability of the pathogen or the vectorial competence, as it can only reflect the fact that the tick took a blood meal on a host infected with this pathogen. Therefore, RNA extraction should be considered as an option to detect live pathogens, which can provide an additional clue when evaluating vectorial competence of ticks when it is not known (Bonnet et al., 2023). Furthermore, the vast majority of viruses transmitted by ticks are RNA viruses, with the exception of viruses of the genus *Asfivirus*, like the *African swine fever virus*. Thus, tick-borne viruses require RNA extraction. As RNA is less stable than DNA, samples should be processed on ice and a reverse-transcription step is generally done in order to detect pathogens on cDNA samples.

DNA extraction can be achieved by chemical extraction using phenol-chloroform (Köchl et al., 2005; Reifenberger et al., 2022), cetyltrimethylammonium bromide (Roux et al., 1996; Reifenberger et al., 2022) or NH_4_OH, which is nevertheless not recommended on larvae and nymphs due to their small size leading to insufficient amount of DNA extracted (Okeyo et al., 2019). However, DNA or RNA extraction is more generally performed using manufacturer kits. Many of them have been tested and evaluated on unfed ticks. For example, Ammazzalorso et al. (2015) showed that DNeasy Blood & Tissue Kit (Qiagen, Valencia, California, USA) and GeneJET Genomic DNA Purification Kit (Thermo Scientific, Waltham, Massachusetts, USA) gave the best results among several tested kits. As blood from engorged ticks can inhibit enzymatic reactions used for the detection of pathogens, extraction steps should remove the vertebrate proteins of the bloodmeal. Reifenberger et al. (2022) showed that phenol-chloroform is best for the extraction of DNA from engorged ticks, whereas the DNeasy Blood & Tissue Kit, despite resulting in lower purity, can be also successfully used for engorged ticks. It is noteworthy that dedicated sequential extraction of both DNA and RNA from the same tick can be done using TRIzol LS Reagent (Cafiso et al., 2021), or Qiagen Virus MinElute kit (Crowder et al., 2010). Extraction of large DNA fragments was also successfully performed from tick samples by multiple mix of phenol:chloroform:isoamylalcohol (Hill and Gutierrez, 2003).

The extraction step is also an important step with respect to issues of contamination. Lejal et al. (2020) have shown that bacterial Operational Taxonomic Units (OTU) detected in ticks were also detected in negative control samples and that more than 50% of the total sequence counts were classified as contaminant bacterial OTU, which have an impact on the microbial diversity of tick sample. This contamination especially occurs during the extraction step done by various manufacturer kits (Lejal et al., 2020). Low biomass samples such as single nymphs are very sensitive to contamination. Careful handling in dedicated areas in the laboratory, cleaning steps of ticks and materials with adequate reagents, or automated extraction methods can lower the contamination level.

## 5 Tick-borne pathogen detection

As TBP nucleic acids are mixed with a majority of tick nucleic acids, or even vertebrate host nucleic acids in the case of engorged ticks, sensitivity and specificity of the detection method used to detect them are really essential.

Nowadays, detection of nucleic acids is still routinely made by PCR directly for DNA samples or following a reverse-transcription step for RNA samples: it is a conventional, cost-effective method, providing rapid results for single pathogen detection by the use of specific designed primers (Ghodrati et al., 2024). Nested PCR (a PCR amplifying a smaller fragment of DNA generated after a first PCR made on a larger fragment) or quantitative PCR (qPCR, which is giving a quantitative value of the DNA template) have been also widely used to improve the specificity or the sensibility of the detection of some bacteria, parasites or viruses (Bhatia and Baersch, 2024; Cicculli et al., 2024; Ghodrati et al., 2024). In each case, the primers used can allow the amplification of a specific species, a whole genus or a group of pathogens. In almost all cases, sequencing is then performed to confirm the result, identify the species or strain, or to conduct phylogenetic studies (Obaid et al., 2024). However, in the case of co-infection, if the primers used can target different sequences, which will then be amplified simultaneously, the sequencing results will be uninterpretable.

In order to detect co-infection, multiple specific PCR (Reis et al., 2011), or multiplex PCR (consisting of a mix of primers applied on a single template, allowing to detect at least two pathogens during the same PCR cycle) (Cardenas-Cadena et al., 2023) can be performed on the same sample. Reverse line blot (RLB) hybridization, a technique using DNA:DNA hybridization with multiple probes, is also used to detect multiple pathogens simultaneously and can also be used to distinguish between strains of the same pathogenic agent within co-infections (Glass et al., 2023). Moreover, microfluidic chips have been developed to target simultaneously several pathogens in the same sample and test several samples at the same time: in a single run, up to 96 TBP can be targeted simultaneously on 96 samples, allowing screening of a large batch of ticks (Moutailler and Galon, 2024). Diaz-Corona et al. (2024) have succeeded in the detection of five different pathogens in *Rhipicephalus sanguineus* ticks by this technique. However, Bernard et al. (2024) reported some limitations of the technique. In their study, detection was done by the chip on a large number of ticks and positive results were confirmed on a smaller number of ticks by PCR and sequencing. Mismatches were observed between positive ticks by species-specific and corresponding genus-specific PCR, in both chips and confirmation PCR (Bernard et al., 2024).

Next-generation sequencing (NGS) is a non-targeted method with which all nucleic acids present in the sample are amplified and analysed, and is still very recently successfully used to detect TBP in ticks (Duan et al., 2024; Intirach et al., 2024; Osikowicz et al., 2024). This technique is easy to employ but in-depth analysis of sequences obtained is crucial. Sequences from the tick host, endosymbionts and microbiota of the tick should be removed from the analysis to identify TBP. In the case of ticks, only the *Ixodes scapularis* genome is known to be complete and can be subtracted from NGS analysis (Osikowicz et al., 2024). One way to overcome the lack of a known genome for other tick species, is to subtract the genome of noninfected laboratory-reared ticks from the same species from sequences from field-collected ticks (Bonnet et al., 2017).

Illumina NovaSeq technology has been also used for the detection of TBP within ticks, clustering by OTU or after a step of bacterial *16S* rRNA amplification and followed by PCR confirmation (Duan et al., 2024; Intirach et al., 2024). Moreover, Osikowicz et al. (2024) have updated a NGS assay combining two Illumina primer mixes of multiplex PCR amplicon sequencing, resulting in the simultaneous identification of tick species and associated TBP during the same reaction.

Sanger sequencing of full-length *16S* rDNA, 454-pyrosequencing, Ion torrent, Illumina-based sequencing of *16S* rDNA hypervariable regions, as well as a whole genome shotgun have been also used to characterize the microbiomes of various tick species and these non-targeted methods also allow the detection of TBPs as reviewed in Bonnet and Pollet (2021).

Finally, it is worth mentioning that antigen-capture ELISA was successfully used to detect *Crimean-Congo haemorrhagic fever virus* in collected ticks in Turkey, using a dedicated manufacturer kit (Yesilbag et al., 2013).

## 6 Emerging methods for TBP detection

New promising tools that are fast and easy to deploy in the field may be apply to TBP detection, but they remain either to be tested on tick samples or developed for more TBP.

Mass spectrometry (MALDI-TOF) has been successfully used for the identification of TBPs from *I. scapularis* samples, including bacteria from the genera *Rickettsia* and *Borrelia* (Smith et al., 2022) or flavivirus such as *deer tick virus* in those ticks (Grant-Klein et al., 2010).

Loop-mediated isothermal (LAMP) is performed at constant temperature, and results are easily interpreted as the reaction contains a colour indicator (Notomi et al., 2000). It has been developed to detect TBP in order to reduce the reaction time, as well as diminish the temperature used. As this technique does not need expensive equipment, it can be carried out in low-resource laboratories. LAMP has been successfully used to detect *Rickettsia rickettsii* (Noden et al., 2018) and *Borrelia burgdorferi* (*s.l.*) (Yang et al., 2013) in field-collected ticks. Even if the *Babesia* sp*.* parasite has been detected by LAMP in vertebrate hosts, it remains to be applied and optimized directly on tick samples (Martínez-García et al., 2021). Since this technique is still under development, there is currently a lack of specific primers and protocols for numerous TBP.

Recombinase polymerase amplification (RPA) is another emerging technique, where the reaction is also operated at a lower temperature than PCR, and results are achieved within 20–30 minutes. Some cultured TBP were successfully detected by RPA (Liu et al., 2016) but no tests on tick samples have yet been published.

Finally, TBDCapSeq, a sequencing assay that uses hybridization capture probes, has successfully covered the complete genomes of 11 TBPs (Jain et al., 2021), and CRISPR/dCas9-mediated biosensor has been used to detect TBP in bacterial culture (Koo et al., 2018). CRISPR/Cas12a has also been combined with RPA in order to detect successfully *Ehrlichia canis* and *Anaplasma platys* in dogs and *Severe fever with thrombocytopenia syndrome virus* in humans (Huang et al., 2022).

## 7 Conclusions

The detection of TBP is essential in the comprehension and the prevention of TBD. In that context, storage, cleaning and grinding of tick samples to be analysed are important steps of due consideration. Nucleic acid extraction methods used to further detect the pathogens need to be chosen regarding the tick life stage and the targeted TBP. For TBP detection, while all kinds of PCR are still widely used, non-targeted methods offer a new range of crucial information in the present context of global changes with significant changes in the distribution of tick populations and associated pathogens. In order to detect potential emergence of new TBP or invasive species, it is, in fact, necessary to carry out increased surveillance without *a priori*. Such surveillance is an essential step to be prepared to develop the most effective possible solutions against TBD. To develop an efficient monitoring, lowering the cost, securing rapid reliable results, as well as limiting the equipment required and developing low resource methods are key points to make the detection of TBP easier in developing countries or in areas of difficult access by using them directly on the field. It is in this direction that research on the detection of TBPs must progress and continue to be supported.

## Funding

This project was supported by the French Government’s Investissement d’Avenir programme, Labex IBEID (ANR-10-LABX-62-IBEID).

## Ethical approval

Not applicable.

## CRediT authorship contribution statement

**Eva Krupa:** Investigation, Writing – original draft, Writing – review & editing. **Alexis Dziedziech:** Writing – review & editing. **Richard Paul:** Writing – review & editing, Supervision, Funding acquisition. **Sarah Bonnet:** Conceptualization, Writing – review & editing, Supervision, Project administration, Funding acquisition.

## Declaration of competing interests

The authors declare that they have no known competing financial interests or personal relationships that could have appeared to influence the work reported in this paper.

## Data Availability

The data supporting the conclusions of this article are included within the article.
